# Supplementary material for: Mapping intracellular dynamics across the whole cell with spatial statistics
Source: Biophys J. 2025 Oct 9;124(23):4205–14. doi: 10.1016/j.bpj.2025.10.005 (PMC12709384; doi:10.1016/j.bpj.2025.10.005)
Supplement: Document S1. Figures S1–S10 and Note S1 [file mmc1.pdf]

Biophysical Journal, Volume *124*

Supplemental information

Mapping intracellular dynamics across the whole cell with spatial statistics

Yohei Okabe, Takumi Saito, Outa Nakashima, Daiki Matsunaga, and Shinji Deguchi

Supporting materials for Okabe et al., "Mapping intracellular dynamics across the whole cell with spatial statistics"

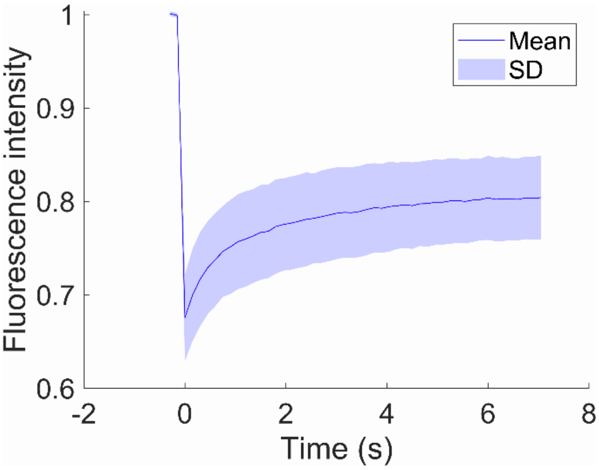

Figure S1. Representative FRAP recovery curve. Fluorescence intensity is normalized to the pre-bleach value ($t = 0$ s). The solid line represents the mean, and the shaded area indicates the standard deviation from $n = 18$ data points within a single cell.

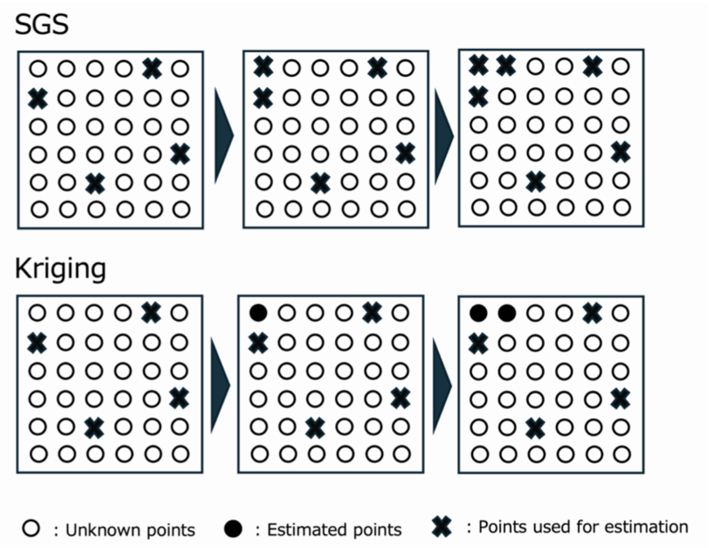

Figure S2 Schematic diagram of SGS and kriging. In SGS, estimation is performed by sequentially incorporating newly estimated data into the known dataset. In contrast, kriging uses only the originally available known data for estimation.

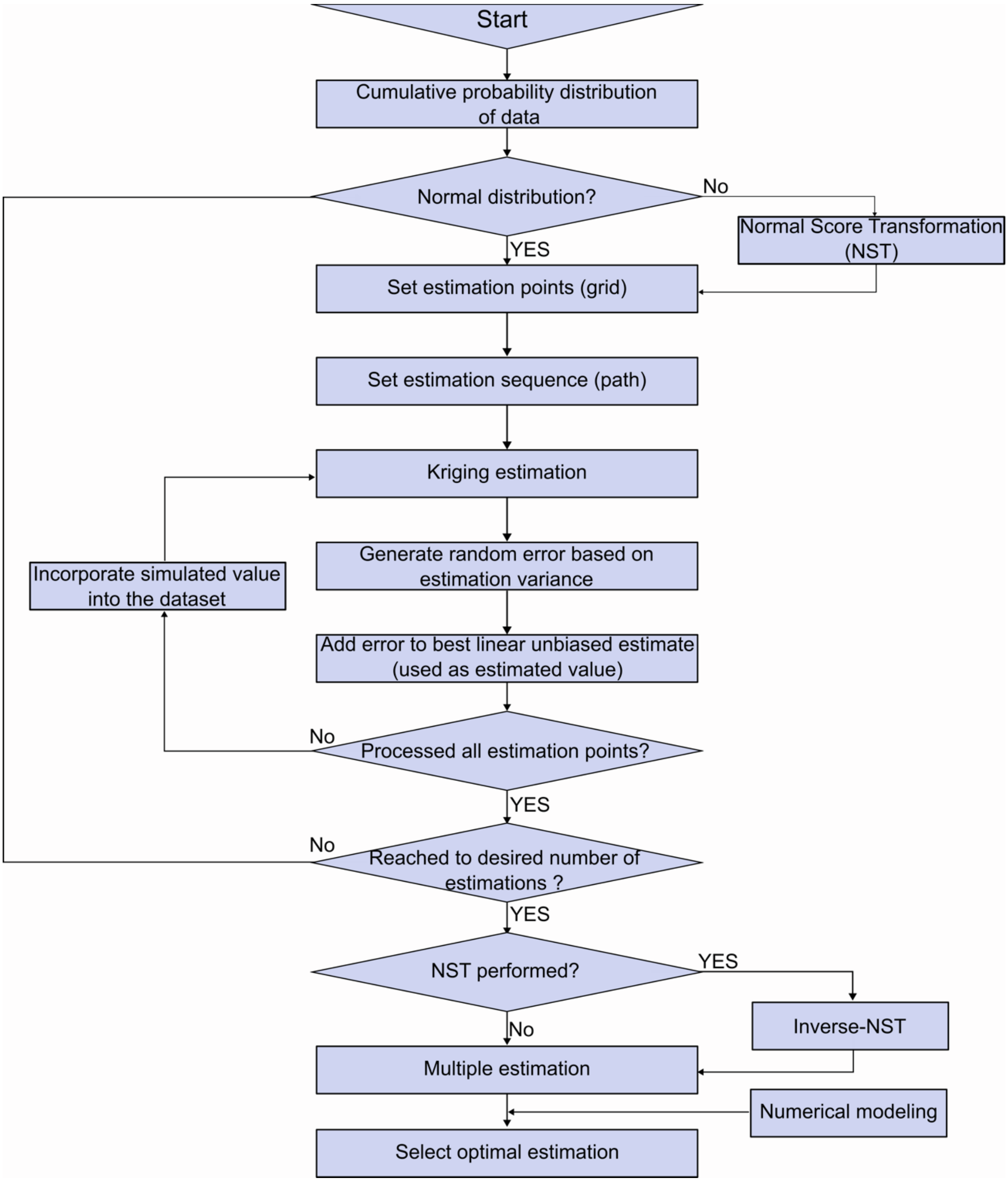

Figure S3 SGS realization used for diffusivity estimation. The diagram illustrates the step-by-step workflow from data transformation and grid setup to sequential kriging with random error incorporation, repeated for multiple realizations to quantify spatial variability.

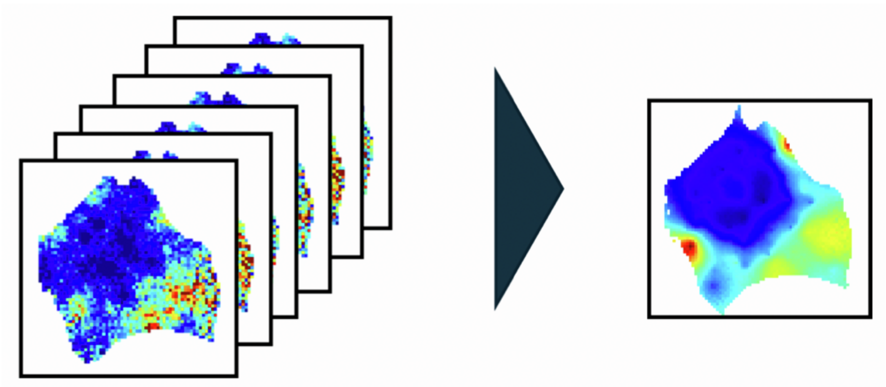

Figure S4 Schematic diagram of SGS simulation averaging. The final estimated value is obtained by averaging the results of multiple simulations.

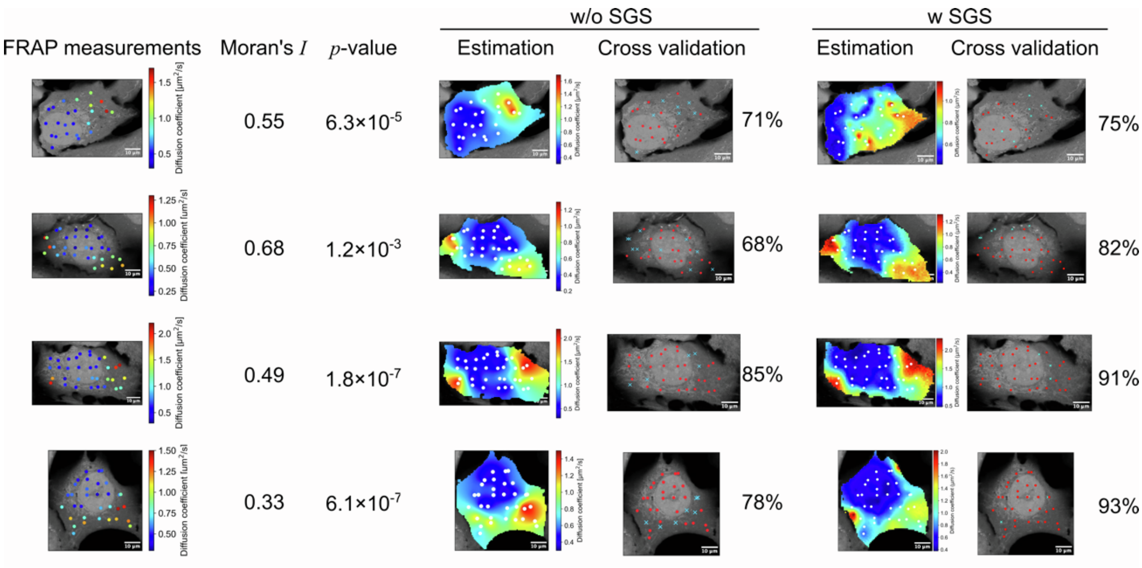

Figure S5 Validation of Pro-FRAP using four representative datasets. The left column shows the diffusion coefficients measured by FRAP, along with the corresponding Moran's I and p-values. The middle column (w/o SGS) and the right column (w SGS) display the diffusion coefficient distributions determined using kriging alone (middle) and Pro-FRAP with SGS (right), respectively. Cross-validation results are also shown, where correctly predicted points are marked in red, and incorrectly predicted points defined as those deviating by more than one standard deviation from the normal distribution are indicated by blue crosses.

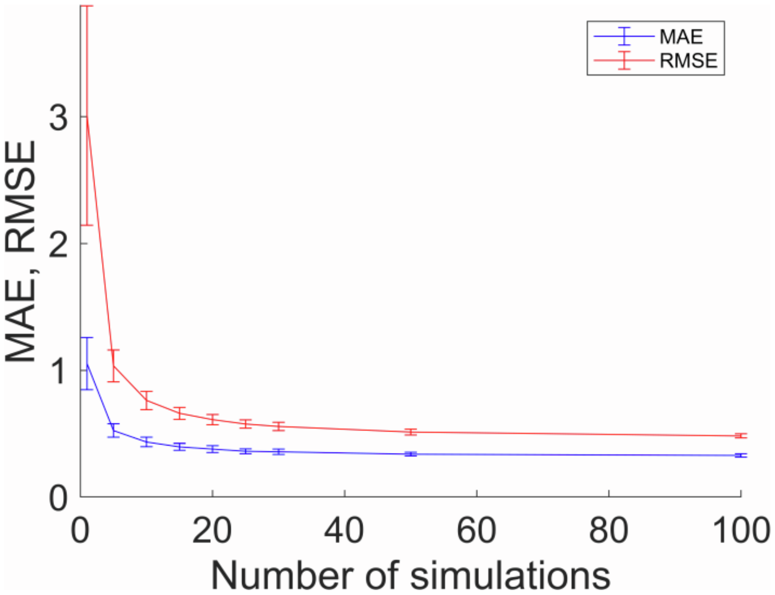

Figure S6 Effect of the number of SGS iterations on error convergence. Both MAE and RMSE sufficiently converge by n = 100.

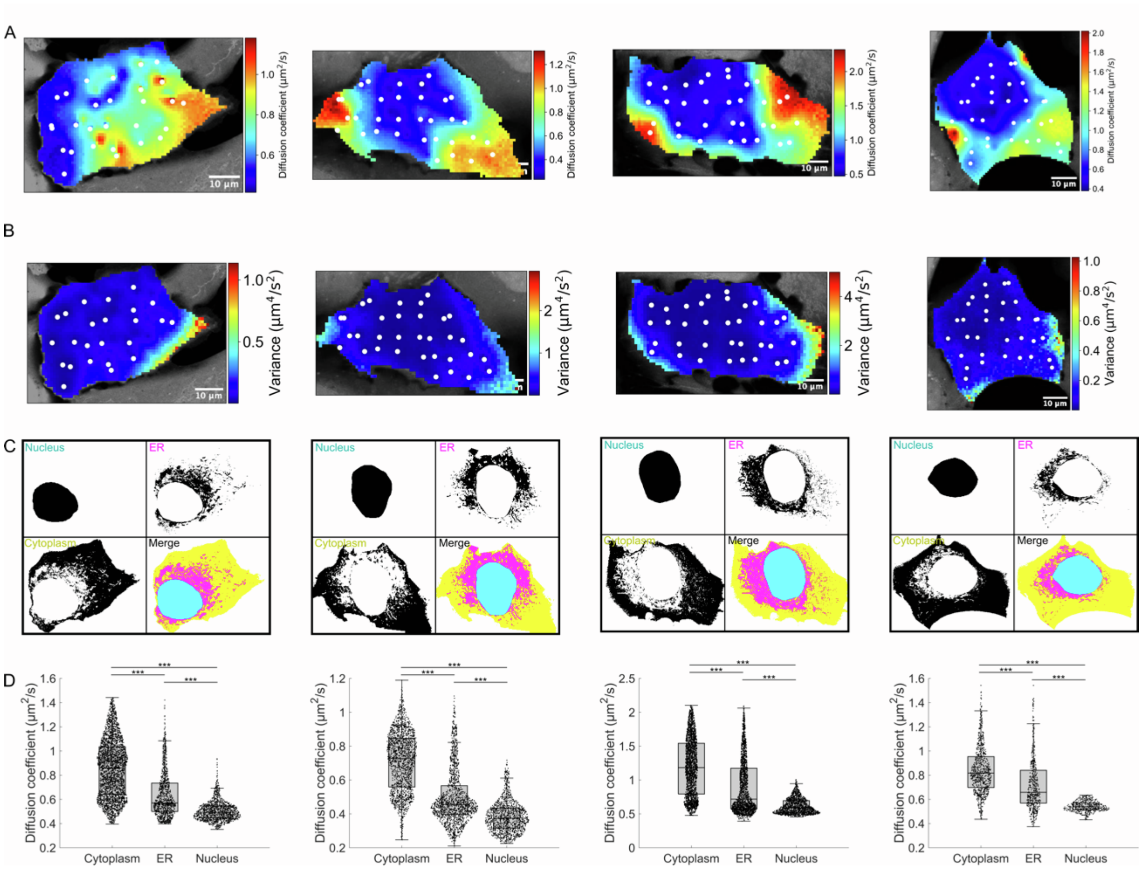

Figure S7 Subcellular analysis of diffusion coefficients for four representative datasets. (A, B) Mean diffusion coefficients (A) and variance (B). (C) Segmentation of subcellular regions into the nucleus, ER, and remaining cytoplasm. (D) Diffusion coefficients for each classified region.

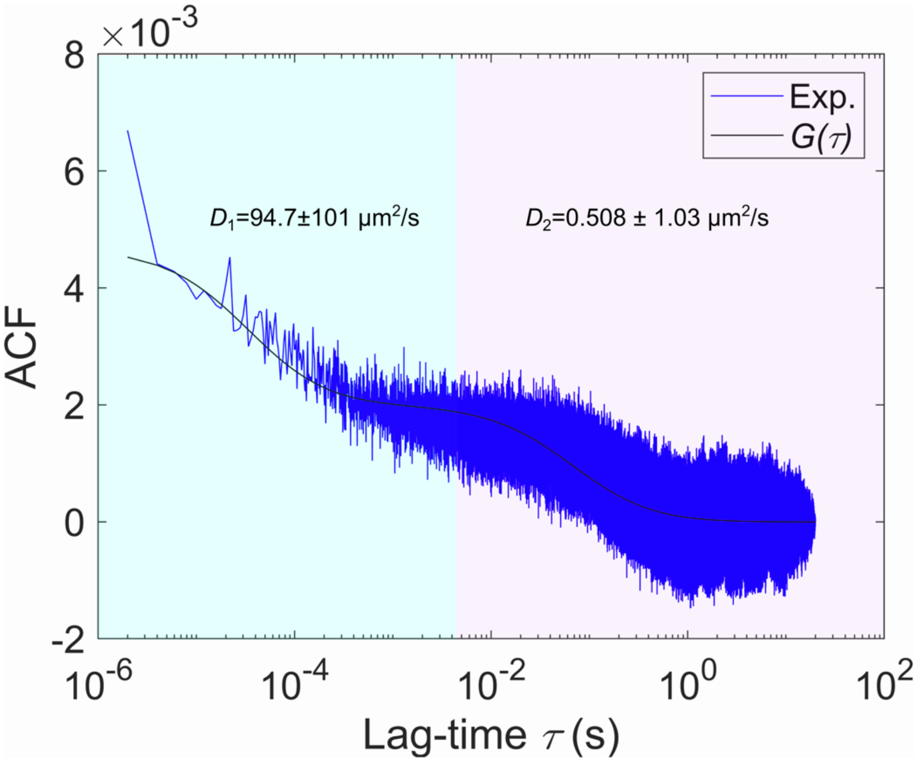

Figure S8 Representative FCS autocorrelation curve and dual-component fitting. ACF derived from a fluorescence intensity time series (blue) with an overlaid fit using a dual-component diffusion model (black line). The fast and slow components correspond to distinct diffusion regimes, as indicated by the shaded cyan and magenta regions, respectively. The slow component is likely due to complex formation following covalent binding of the dye to thiol-containing macromolecules after esterase activation.

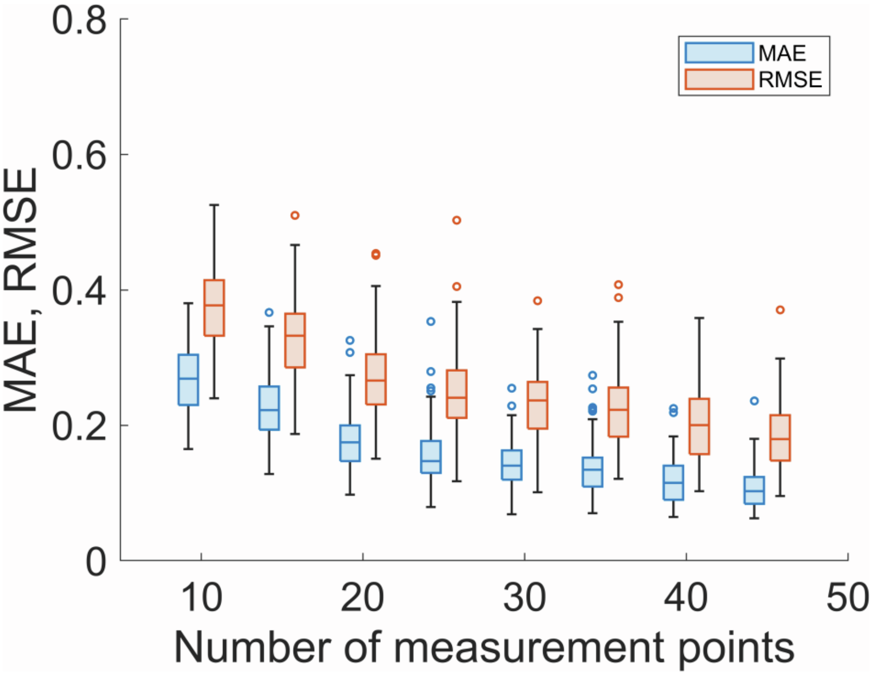

Figure S9 Evaluation of ordinary kriging. Effect of increasing the number of measurement points in ordinary kriging alone on errors (MAE in blue and RMSE in red) when σ/R_{cell} is fixed at 0.56.

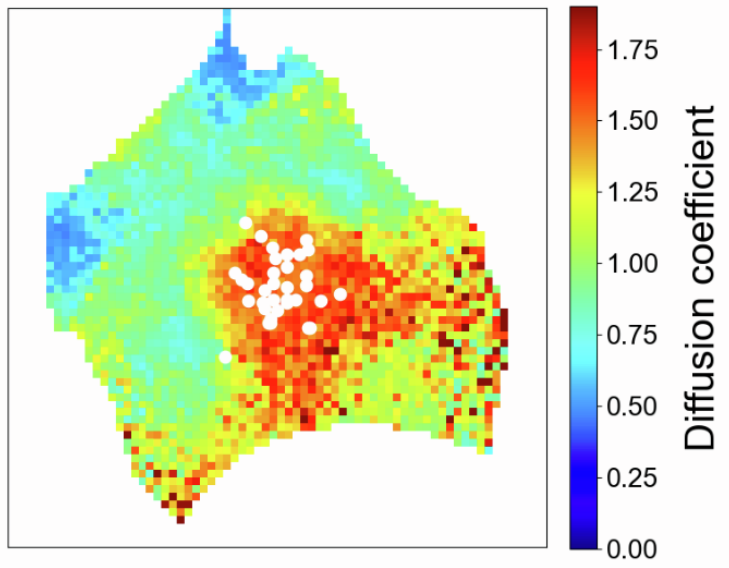

Figure S10 Additional analysis of Fig. 4D. Diffusion coefficients determined by Pro-FRAP with a Gaussian-distributed pattern of σ/R_{cell} = 0.17, corresponding to Fig. 4D.

Supporting note 1

Ordinary kriging assumes a steady state in the spatial domain, and the mean of a physical variable m is constant but unknown. The unbiasedness condition for predictions is expressed as

$$\sum_{k=1}^{n} \omega_k = 1 \qquad \text{(S1)}$$

Here, ω_k represents the weights assigned to the observed data positions u_k. The prediction error variance of a variable Z relative to the true value is expressed as

$$\begin{aligned}
\sigma_\varepsilon^{2*} &= V[\, Z^*(\, u_o) - Z(\, u_o)] \\
&= V[\textstyle\sum_{k=1}^{n} \omega_k\, Z(u_k) - Z(u_o)] \\
&= V\left[\textstyle\sum_{k=1}^{n} \omega_k \left(Z(u_k) - Z(u_o)\right)\right] \\
&= E[\{\textstyle\sum_{k=1}^{n} \omega_k (Z(u_k) - Z(u_o))\}^2] \\
&= \textstyle\sum_{j=1}^{n} \sum_{k=1}^{n} \omega_j \omega_k E\left[\left(Z(u_j) - Z(u_o)\right)\left(Z(u_k) - Z(u_o)\right)\right] \qquad \text{(S2)}
\end{aligned}$$

where V and E represent the variance and expected value, respectively; u_o and u_k denote the positions of an unobserved point and of an arbitrary point, respectively; and an asterisk indicates estimated variables. The semi-variogram is defined as half of the squared difference between values at any given distance h:

$$\gamma(h_{j,k}) = \frac{1}{2} V[Z(u_j) - Z(u_k)] = \frac{1}{2} E\left[\left(Z(u_j) - Z(u_k)\right)^2\right]$$

$$= \frac{1}{2} E\left[\left(Z(u_j) - Z(u_o)\right)^2\right] + \frac{1}{2} E[(Z(u_k) - Z(u_o))^2] - E[(Z(u_j) - Z(u_o))(Z(u_k) - Z(u_o))]$$

$$= \gamma(h_{o,j}) + \gamma(h_{o,k}) - E[(Z(u_j) - Z(u_o))(Z(u_k) - Z(u_o))] \quad .$$

(S3)

Substituting Eq. (S3) into Eq. (S2) and eliminating E,

$$\sigma_{\varepsilon}^{2*} = \sum_{j=1}^{n}\sum_{k=1}^{n}\omega_j\omega_k\left(\gamma(h_{o,j}) + \gamma(h_{o,k}) - \gamma(h_{j,k})\right)$$

$$= \sum_{j=1}^{n}\omega_j\gamma(h_{o,j}) + \sum_{k=1}^{n}\omega_k\gamma(h_{o,k}) - \sum_{j=1}^{n}\sum_{k=1}^{n}\omega_j\omega_k\gamma(h_{j,k})$$

$$= 2\sum_{k=1}^{n}\omega_k\,\gamma(h_{o,k}) - \sum_{j=1}^{n}\sum_{k=1}^{n}\omega_j\omega_k\gamma(h_{j,k}). \qquad \text{(S4)}$$

We consider a Lagrange multiplier problem to minimize the semi-variogram under the constraint given by Eq. (S1),

$$Q = \frac{1}{2}\,\sigma_{\varepsilon}^{2*} + \mu\left(1 - \sum_{k=1}^{n}\omega_k\right). \qquad \text{(S5)}$$

The variables ω_k and μ are determined by minimizing the objective function Q, and thus

$$\frac{\partial Q}{\partial\omega_k} = \gamma(h_{j,o}) - \sum_{k=1}^{n}\omega_k\gamma(h_{j,k}) - \mu = 0 \qquad \text{(S6)}$$

and

$$\frac{\partial Q}{\partial\mu} = 1 - \sum_{k=1}^{n}\omega_k = 0 \qquad \text{(S7)}$$

The combination of Eqs. (S6) and Eq. (S7) forms the kriging equations (i.e., Eq. (3) and (4) in the main text), which are a system of $n+1$ linear equations with unknowns ω_k

$(k = 1, 2, \ldots, n)$ and μ.
