# Supplementary material for: Mapping intracellular dynamics across the whole cell with spatial statistics
Source: Biophys J. 2025 Oct 9;124(23):4205–14. doi: 10.1016/j.bpj.2025.10.005 (PMC12709384; doi:10.1016/j.bpj.2025.10.005)
Supplement: Document S2. Article plus supporting material [file mmc2.pdf]

Biophysical Journal
Article

Biophysical Society

Mapping intracellular dynamics across the whole cell with spatial statistics

Yohei Okabe,[1] Takumi Saito,[1,*] Outa Nakashima,[1] Daiki Matsunaga,[1] and Shinji Deguchi[1,2,3,*]
[1]Graduate School of Engineering Science, The University of Osaka, Toyonaka, Osaka, Japan; [2]R[3] Institute for Newly-Emerging Science Design, The University of Osaka, Toyonaka, Osaka, Japan; and [3]Global Center for Medical Engineering and Informatics, The University of Osaka, Toyonaka, Osaka, Japan

ABSTRACT Understanding molecular diffusion within cells is crucial for gaining insights into cellular biophysical mechanisms. Despite its importance, achieving versatile mapping of such diffusion across whole cells remains challenging, as many live-cell measurement techniques including fluorescence recovery after photobleaching (FRAP) provide data only at discrete spatial locations due to experimental constraints. To overcome this limitation, we developed probabilistic FRAP (Pro-FRAP), a novel approach that integrates FRAP with sequential Gaussian simulation (SGS), an advanced spatial statistical method incorporating probabilistic modeling to estimate diffusion in unmeasured regions. Pro-FRAP applies SGS to standardize measured FRAP data, perform conditional simulations based on spatial correlations, and generate statistically robust estimates. In separate analyses, numerical simulations were conducted to optimize the spatial arrangement of measurement points, enhancing data accuracy and coverage. Unlike deterministic interpolation methods, Pro-FRAP captures spatial variability and quantifies uncertainty in intracellular diffusion, providing a more detailed representation of molecular transport. While this study focuses on molecular diffusion, the proposed approach is applicable to other intracellular dynamics measurable at limited spatial points such as molecular turnover, thus offering a generalizable tool for whole-cell biophysical analysis under sparse sampling conditions.

SIGNIFICANCE Measuring intracellular dynamics at high spatial resolution is critical for understanding how cells function. However, many live-cell measurement techniques, such as conventional fluorescence recovery after photobleaching (FRAP), are limited to sparse spatial points due to experimental constraints. We introduce probabilistic FRAP (Pro-FRAP), a framework that integrates local measurements with spatial modeling to estimate dynamic properties across entire cells with improved statistical robustness. This approach is potentially applicable to a wide range of biophysical measurements and offers a versatile tool for whole-cell analysis under sparse sampling conditions.

INTRODUCTION

Understanding intracellular dynamics is crucial for gaining insight into the complex biophysical mechanisms that maintain cellular homeostasis. Processes such as molecular diffusion facilitate the transport of signaling molecules, nutrients, and other essential compounds, ensuring cellular functionality and adaptability. Probing these dynamics advances our comprehension of cellular physiology, stress responses, disease mechanisms, and therapeutic effects.

Fluorescence recovery after photobleaching (FRAP) is a powerful technique for evaluating molecular dynamics, particularly diffusion, in living cells (1–5). In FRAP measurements, defined regions of a cell are photobleached, and the subsequent recovery of fluorescence is monitored over time. This recovery indicates the movement of fluorescently labeled molecules within the bleached region, allowing for the quantification of diffusion coefficients. FRAP has been pivotal in revealing intracellular dynamics, providing quantitative insights into the local mobility and functional roles of molecules. Its broad applicability across different cell types and organisms (6,7) has made FRAP an indispensable tool in biology research.

Despite its significant contributions, FRAP is inherently limited by its localized measurement approach, which poses challenges for whole-cell analysis. Specifically, conducting FRAP measurements at multiple sites within a single cell can lead to excessive photobleaching, reducing overall fluorescence intensity and therefore compromising measurement

Submitted February 8, 2025, and accepted for publication October 3, 2025.
*Correspondence: saito.takumi.es@osaka-u.ac.jp or deguchi.shinji.es@osaka-u.ac.jp
Editor: Rumiana Dimova.
https://doi.org/10.1016/j.bpj.2025.10.005
© 2025 The Author(s). Published by Elsevier Inc. on behalf of Biophysical Society.
This is an open access article under the CC BY license (http://creativecommons.org/licenses/by/4.0/).

accuracy. This practical constraint confines FRAP to discrete regions, limiting the acquisition of comprehensive spatial data across the entire cell. Consequently, capturing the full complexity of molecular dynamics within the cellular context becomes difficult, preventing a thorough analysis. This limitation is particularly significant when studying processes that involve extensive spatial variation, such as intracellular transport and membrane trafficking, where understanding the global behavior is crucial.

To address these limitations, we propose probabilistic FRAP (Pro-FRAP), a novel approach that integrates FRAP with sequential Gaussian simulation (SGS), an advanced spatial statistical method, to estimate molecular diffusion across unmeasured regions. Unlike deterministic methods, such as interpolation through averaging between measurement points (8,9), SGS is a probabilistic simulation technique that generates multiple realizations incorporating spatial variability, enabling an uncertainty-aware assessment of diffusion patterns (10–12). In this study, we focus on molecular diffusion as a representative application and demonstrate how Pro-FRAP can extend conventional diffusion analysis beyond localized measurements, providing statistically robust and spatially continuous estimates of the intracellular diffusion landscape. Importantly, although exemplified here using FRAP-based diffusion analysis, the probabilistic framework itself can be applied to various sparse biophysical measurements, particularly when aiming to achieve whole-cell-level mapping from limited measurements. It complements existing techniques to reconstruct spatial heterogeneity in cellular properties.

MATERIAL AND METHODS

Cell culture

U2OS cells (HTB-96, ATCC) were cultured in high-glucose DMEM (Wako, FUJIFILM Wako Pure Chemical, Osaka, Japan) supplemented with 10% fetal bovine serum (Sigma, Burlington, MA, USA), 1% penicillin-streptomycin (Wako), and 200 mM L-glutamine, and maintained at 37°C in a humidified 5% CO_2 incubator. Fluorescent probes, 5-chloromethylfluorescein diacetate (CellTracker-Green, Invitrogen, Carlsbad, CA, USA), were diluted to 1 mM with DMSO to prepare a stock solution. Cells were incubated in Opti-MEM containing 5-chloromethylfluorescein diacetate at a final concentration of 5 μM for 30 min at 37°C. For experiments, cells were incubated in FluoroBright (Gibco, Waltham, MA, USA) supplemented with 1 M HEPES, 10% fetal bovine serum (Sigma), 1% penicillin-streptomycin (Wako), 200 mM L-glutamine, and 100 mM sodium pyruvate.

FRAP experiments

FRAP experiments were performed with a confocal laser scanning microscope (FV3000, Olympus, Center Valley, PA, USA) mounting a 60×/NA 1.42 objective. To increase the frame rate, the entire image of a single cell was divided into four segments, which were sequentially captured. Within each segment, images were acquired using a 488-nm excitation laser, and multiple circular regions spaced ∼10 μm apart were photobleached using 405- and 488-nm lasers. Images were acquired every 150–200 ms, with 15–20 frames collected per measurement. The first two frames were acquired before bleaching, followed by a 150–200-ms bleaching phase during which 3–5 circular bleach regions (approximately 3 μm in diameter) were simultaneously targeted. Each spot was bleached only once without repeated exposure. Recovery imaging began from the third frame and continued for 13–18 frames, during which the recovery curve typically reached a plateau (Fig. S1). To correct for overall photobleaching during acquisition, we applied the Bleach Correction plugin in Fiji/ImageJ software (National Institutes of Health), using fluorescence intensity in non-bleached regions as a reference. The temporal evolution of fluorescence intensity distribution within each circular region $F(r,t)$ was modeled with a Gaussian function

$$F(r,t) = A_1 - \frac{A_2}{4Dt+\rho^2}\exp\left(-\frac{r^2}{4Dt+\rho^2}\right) \quad (1)$$

where D is the diffusion coefficient, and A_1, A_2, and ρ are fitting parameters, all determined using the least-squares method (6,13,14).

Ordinary kriging

To estimate spatially distributed D values from measured data points, ordinary kriging was employed as a spatial statistics-based interpolation method (15). This approach provides optimal linear unbiased predictions by incorporating the spatial autocorrelation of the data to estimate unknown values with minimal error. Specifically, ordinary kriging utilizes a semivariogram to quantify how spatial variance varies with the distance between data points, enabling statistically robust interpolation even in heterogeneous datasets. The predicted value at an unobserved location o is expressed as a linear combination of values observed at locations k,

$$Z^*(u_o) = \sum_{k=1}^{n}\omega_k Z(u_k) \quad (2)$$

where the kriging weights ω_k represent the contributions of each observed value Z at position u_k, u_o denotes the position of an unobserved point, an asterisk indicates estimated variables, and n is the total number of observed data points. The weights ω_k are determined by solving the following kriging equations,

$$\sum_{k=1}^{n}\omega_k\gamma\left(h_{j,k}\right)+\mu = \gamma\left(h_{j,o}\right);\ j = 1,2,\ldots,n \quad (3)$$

$$\sum_{k=1}^{n}\omega_k = 1 \quad (4)$$

where the terms $\gamma(h_{j,k})$ and $\gamma(h_{j,o})$ are the semivariogram for the distance between observation positions u_j and u_k and between observation positions u_j and the prediction position u_o, respectively, and the parameter μ is the Lagrange multiplier ensuring unbiased predictions (see Note S1 for details). The semivariogram itself is calculated directly from the raw data of the FRAP experiments Z,

$$\gamma\left(h_{i,j}\right) = \frac{1}{2}\left(Z(u_i) - Z\left(u_j\right)\right)^2 \quad (5)$$

and is characterized by fitting a widely used exponential model to the variogram cloud, consisting of all data,

$$\gamma(h) = \begin{cases} b+(c-b)\left\{1-\exp\left(-\frac{h}{a}\right)\right\} & \text{if } h>0 \\ 0 & \text{otherwise} \end{cases} \quad (6)$$

where a, b, and c denote the correlation length, nugget, and sill, respectively. By minimizing the prediction variance $\frac{1}{2}\sigma_\varepsilon^{2*}$, where the factor 1/2 is included to simplify quadratic terms, ordinary kriging ensures the best

linear unbiased prediction while considering spatial autocorrelation and measurement uncertainty. Solving the resulting kriging equations provides both the predicted values and the associated prediction uncertainty.

Spatial autocorrelation analysis

Moran's I, defined as

$$I = \frac{n}{S_0} \frac{\sum_{i=1}^{n} \sum_{j=1}^{n} W_{ij}(Z_i - \overline{Z})(Z_j - \overline{Z})}{\sum_{i=1}^{n} (Z_i - \overline{Z})} \tag{7}$$

was employed to evaluate the spatial autocorrelation of datasets, ensuring the robustness of the interpolation results, in which $\overline{Z}$ is the mean of the data, and W_{ij} represents the spatial weight between data points i and j. W_{ij} and S_0 are defined as

$$W_{ij} = \begin{cases} d_{ij}^{-2} & d_{ij} \le \overline{d} \\ 0 & d_{ij} > \overline{d} \end{cases} \tag{8}$$

and

$$S_0 = \sum_{i=1}^{n} \sum_{j=1}^{n} W_{ij} \tag{9}$$

respectively, where d_{ij} is the distance between u_i and u_j, and $\overline{d}$ is the mean of d_{ij}. To statistically test the spatial correlation, the z-score is defined as

$$z_I = \frac{I - E[I]}{\sqrt{E[I^2] - E[I]^2}} \tag{10}$$

where E represents the expected value. The null hypothesis assuming no spatial autocorrelation is tested using the p value derived from the z-score and the standard normal distribution. A p value less than 0.01 was considered statistically significant, indicating the presence of spatial autocorrelation in the dataset.

Sequential Gaussian simulation

Pro-FRAP utilizes SGS, a probabilistic statistical method (10–12) used to generate spatially distributed values that preserve spatial variability while closely matching observed data, effectively creating maps that agree with the measurements while retaining the natural irregularities found in real spatial patterns. While kriging provides a single optimal estimate based on spatial interpolation, SGS employs kriging as part of a stochastic simulation process, generating multiple realizations to represent plausible spatial distributions and quantify the uncertainty in these predictions (Fig. S2). To ensure the validity of the statistical framework, we confirmed that the core conditions commonly required in standard spatial modeling were satisfied to a reasonable degree (Fig. S3). These conditions help ensure that the simulation behaves in a statistically consistent way with the observed data: 1) the data distribution was approximately normal, achieved by applying a normal score transformation, to ensure values follow a distribution suitable for kriging; 2) the data exhibited spatial continuity, meaning that nearby locations tended to have similar values, capturing the tendency for spatially close points to share similar measurements, as characterized by empirical variograms; and 3) local conditioning was applied so that each new simulated value was influenced by nearby measured values, ensuring that local patterns in the observed data are preserved, implemented through sequential kriging with its associated conditional variance. To reduce computational load, the original data captured at a spatial resolution of 300 × 300 pixels was downsampled to 70 × 70 pixels using a moving average before computation. A grid for estimation points was then constructed based on the observed cell morphology, restricting the estimation to intracellular regions. The sequence of estimation points, which strongly influences the simulation outcome, was determined randomly using uniform random numbers, to minimize systematic biases that could arise from a fixed ordering. At each estimation point, ordinary kriging was used to compute the best linear unbiased estimate of the value at the location, along with its corresponding error variance. To reflect this uncertainty and avoid producing an overly smooth map, a random value drawn from a normal distribution with variance equal to the kriging error variance was then added to the estimate to account for spatial uncertainty. These estimated values were sequentially incorporated into the dataset, ensuring that each subsequent estimation was based on the updated dataset that included all previously estimated values, thus preserving spatial consistency. For datasets that had undergone normal score transformation, an inverse transformation was applied at the final stage to return the estimated values to their original scale. The simulation process was repeated 100 times within the Pro-FRAP framework, generating multiple realizations for each estimation point (Fig. S4). The mean of these realizations was taken as the final predicted value, and their spread was used as a measure of spatial variability and the confidence in the predictions.

Analysis of intracellular diffusion coefficients

The intracellular diffusion coefficients were analyzed based on the fluorescence intensity of 5-chloromethylfluorescein diacetate, which reflects the degree of probe uptake. To achieve this, thresholding was applied using Fiji/ImageJ software to automatically segment and extract regions. Intracellular regions were classified into the nucleoplasm, endoplasmic reticulum (ER), and the rest of the cytoplasm. Data were collected from $N = 4$ independent experiments, and statistical comparisons of diffusion coefficients across categories were performed. Statistical significance was assessed using an unpaired two-tailed Student's t-test for raw experimental data before interpolation and using a one-way analysis of variance for data after interpolation. Statistical significance was defined as follows: $***p \le 0.001$.

Simulated evaluation of spatial sampling strategies

To identify optimal spatial sampling patterns for accurately estimating intracellular diffusion coefficients, we simulated interpolation processes across multiple spatial configurations. The analysis considered three key factors: 1) the spatial arrangement of measurement points, 2) the underlying diffusion coefficient distribution, and 3) cellular morphology. First, two spatial sampling patterns were examined: a Gaussian-distributed pattern, where photobleached positions were assigned based on a Gaussian function with varying standard deviations σ centered within the cell, and a lattice-based pattern, where measurement points were arranged in a grid-like structure. Second, diffusion coefficient distributions were modeled in two ways: a radial gradient with concentric contours, where diffusion coefficients gradually changed with distance from the center, and a stepwise variation, where diffusion coefficients exhibited discrete jumps between concentric regions rather than a smooth gradient. Third, two distinct cellular morphologies, derived from experimental images, were analyzed to assess the impact of cell shape on prediction accuracy. To evaluate the influence of these factors, interpolated diffusion maps generated by Pro-FRAP were compared against the original ground truth using two error metrics: mean absolute error (MAE) and root mean-squared error (RMSE).

Fluorescence correlation spectroscopy

Fluorescence correlation spectroscopy (FCS) experiments were conducted by a point scanning mode of the confocal laser scanning microscope

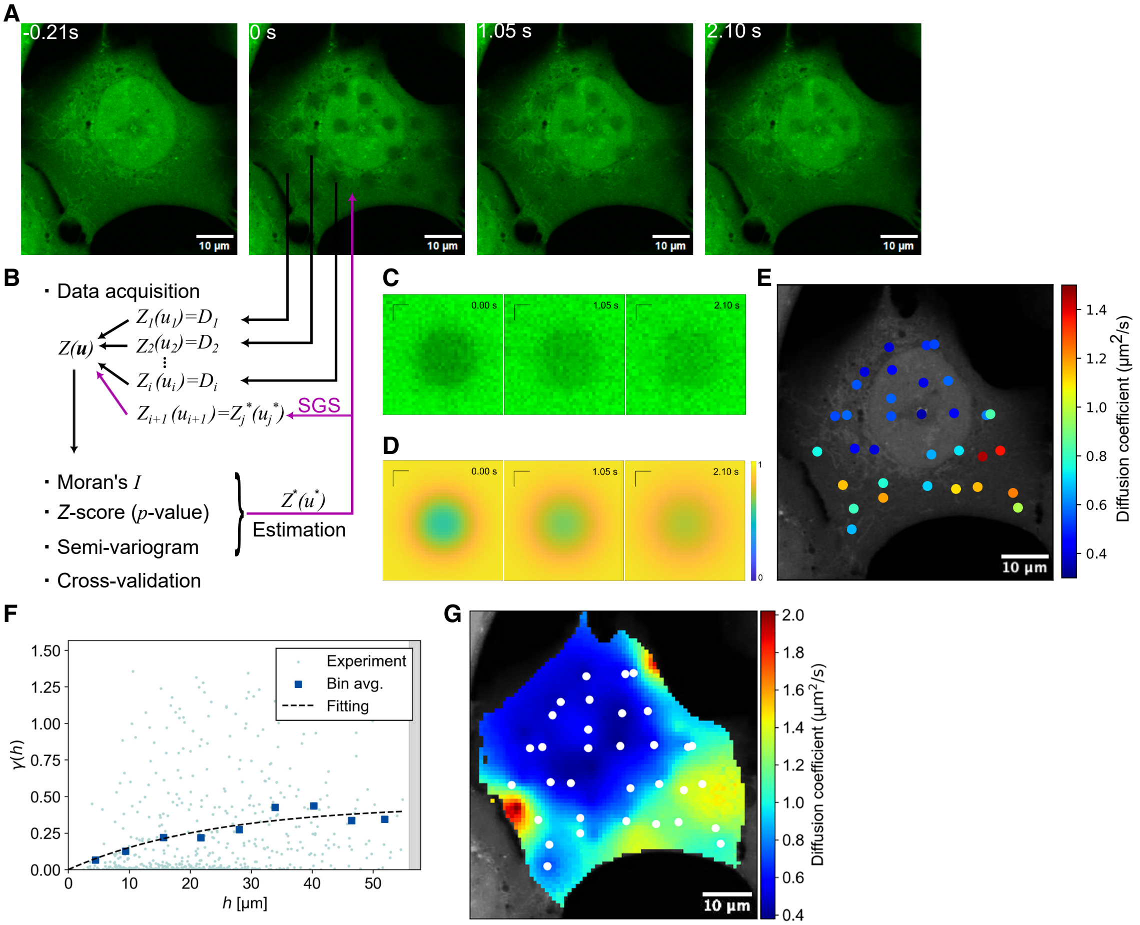

FIGURE 1 Demonstration of Pro-FRAP. (*A*) FRAP experiments on fluorescent probes, 5-chloromethylfluorescein diacetate in a U2OS cell. (*B*) Schematics of workflow in spatial statics. (*C* and *D*) Time course of a representative FRAP region in experiments (*C*) and regression analysis (*D*). (*E*) Diffusion coefficients measured at each FRAP region. (*F*) The semivariogram of each data pair (*blue dots*), data binning (*blue rectangle*), and a regression curve (*dashed line*). (*G*) Diffusion coefficients determined by Pro-FRAP. White dots represent measurement points. Scale bars, 1 μm (*C* and *D*) and 10 μm (*A*, *B*, and *E–G*).

(FV3000, Olympus) mounting the 60×/NA 1.42 objective. Measurement points were randomly selected in cells containing 5-chloromethylfluorescein diacetate. Fluorescence signal $F(t)$ was acquired every 2 μs for a duration of 20 s. The autocorrelation function (ACF) was obtained by

$$ACF(\tau) = \frac{\langle \delta F(t) \delta F(t+\tau) \rangle}{\langle F(t) \rangle^2}. \tag{11}$$

Diffusion coefficients were determined by fitting ACF to a dual-component diffusion model expressed as

$$\begin{aligned} G(\tau) = & N_1^{-1}\left(1+\frac{\tau}{\tau_{D1}}\right)^{-1}\left(1+\frac{\tau}{s^2\tau_{D1}}\right)^{-\frac{1}{2}} \\ & + N_2^{-1}\left(1+\frac{\tau}{\tau_{D2}}\right)^{-1}\left(1+\frac{\tau}{s^2\tau_{D2}}\right)^{-\frac{1}{2}}, \end{aligned} \tag{12}$$

$$D_1 = \frac{w_{xy}^2}{4\tau_{D1}}, D_2 = \frac{w_{xy}^2}{4\tau_{D2}} \tag{13}$$

where N_i and τ_{Di} are the number of photons and the characteristic time of i-th components ($i = 1$ or 2), and w_{xy} and S are imaging parameters that depend on the microscopic setup. Using the known diffusion coefficient of the fluorophore rhodamine B, $w_{xy} = 0.21\mu$m and $S = 2.67$ were calibrated.

RESULTS

Whole-cell diffusion mapping with Pro-FRAP

As a representative application of our spatial statistical framework, we conducted whole-cell diffusion mapping in U2OS cells using the fluorescent probe 5-chloromethylfluorescein diacetate (Fig. 1 *A*). Photobleaching was performed at multiple sites approximately equidistantly distributed throughout individual cells. To determine the diffusion coefficients in each photobleached region, spatial and temporal intensity changes were analyzed using the least-squares method with a Gaussian model (Fig. 1, *B–D*). The diffusion coefficients were then obtained as a discrete set of values (Fig. 1 *E*) and subsequently utilized as a dataset to calculate Moran's *I*, a measure of spatial correlation. Using a spatial weights matrix to define

relationships between data points based on their spatial proximity, the associated p value was determined by comparing the observed Moran's I to its expected value under the null hypothesis of no spatial correlation (Fig. 1 *B*). A semivariogram was constructed to represent variance as a function of distance, and a widely used exponential model was fitted to determine the spatial structure parameters (Fig. 1 *F*) (15). The parameter a in Eq. 6, referred to as the "range" in the exponential model and representing the distance within which spatial correlation is significant, was found in many cases to exceed the characteristic length scale of individual cells, i.e., ~30–50 μm, indicating that spatial correlation was present within the cell. Subsequently, diffusion coefficients at each pixel within cells were determined through the process of SGS (Fig. 1 *G*). Many of these datasets exhibited spatial correlation, as indicated by high Moran's I values (>0.3) and low p values (<0.001) (Fig. S5). These results indicate that the statistical reliability of the estimated diffusion maps is supported by the strong spatial correlation.

To evaluate the effectiveness of our approach, we conducted a cross-validation analysis (Fig. S5). While ordinary kriging analytically provides a single optimal estimate and is computationally straightforward, SGS repeatedly performs kriging to generate multiple realizations, enabling explicit quantification of spatial uncertainty. Therefore, for approximate mapping where uncertainty quantification is not critical, ordinary kriging can be sufficient, whereas SGS is preferable when evaluating local uncertainty and reproducing spatial variability. Cross-validation is an essential step in assessing the validity and reliability of predictions. By dividing the data into subsets for training and testing, this method ensures that predictions are evaluated using unseen data, providing an unbiased estimate of model performance and minimizing the risk of overfitting. The analysis showed that the SGS-based approach consistently yielded more accurate estimates than ordinary kriging alone, a widely used method in conventional spatial statistics, across all four representative datasets. Specifically, the prediction accuracies for ordinary kriging alone were 71, 68, 85, and 78%, while the incorporation of SGS improved these values to 75, 82, 91, and 93%, respectively, demonstrating that SGS enhances predictive performance by effectively capturing spatial variability. In separate analyses, we evaluated the effect of repeated SGS simulations and confirmed that the results sufficiently converged by $n = 100$, stabilizing within a well-defined range of uncertainty (Fig. S6). Examining the spatial distribution of this uncertainty, variance remained higher around the cell periphery, likely due to the lower density of measurement points, making extrapolation challenging (Fig. S7). Nevertheless, the above strong spatial correlation, robust cross-validation performance, and the convergence of SGS estimates justify the use of Pro-FRAP, which applies spatial statistics to estimate unknown diffusion coefficients with statistical rigor.

The relatively low diffusion coefficients estimated here (~0.5–2 $\mu m^2/s$) reflect the behavior of 5-chloromethylfluorescein diacetate after intracellular processing. Upon activation by intracellular esterases, the dye is known to covalently bind to thiol-containing macromolecules, thereby reflecting the diffusivity of protein complexes. This interpretation is supported by our FRAP results, which show partial recovery with an immobile fraction (Fig. S1). Consistent with this, FCS measurements in the same cell type revealed both fast and slow components (Fig. S8). The slow component matches the FRAP-derived values, whereas the fast component likely reflects freely diffusing molecules that fall outside the temporal resolution of our FRAP setup.

Spatial analysis of intracellular diffusion coefficients

Four representative raw datasets (Fig. S5), obtained by conventional FRAP measurements at discrete points without spatial statistical processing, were analyzed for subcellular analysis of diffusion coefficients. In all cases, the diffusion coefficients were significantly higher in the cytoplasm compared with the nucleoplasm, with the measurements selectively performed at individually chosen locations during experiments (Fig. 2, *A* and *B*). In contrast, SGS was applied to estimate the diffusion coefficient as a probabilistic variable across the entire cell, using only the data obtained from these same measurement points, resulting in a mapped representation of the mean values (Fig. 2 *C*). For the same representative datasets, regions were classified into the nucleoplasm, the ER, and the rest of the cytoplasm based on the fluorescence intensity of 5-chloromethylfluorescein diacetate, which reflects the extent of probe uptake (Fig. 2 *D*). In all datasets, the diffusion coefficients were highest in the cytoplasm, intermediate in the ER, and lowest in the nucleoplasm (Fig. 2 *E*), a trend consistently observed in individual cells (Fig. S7). These results demonstrate that, while traditional FRAP measurements are limited to specific sampling points (Fig. 2, *A* and *B*), Pro-FRAP provides statistically robust diffusion maps that align with trends observed at individual measurement points (Fig. 2, *C* and *E*). This novel approach, therefore, allows for the inference of region-specific characteristics within cells even when the measurement points are limited.

Numerical simulations evaluate FRAP sampling strategies

The accuracy of intracellular diffusion mapping may be influenced by both the spatial arrangement of measurement points and the underlying diffusion distribution. To systematically investigate these potential factors, numerical simulations were conducted using SGS-based diffusion mapping, incorporating different FRAP sampling patterns

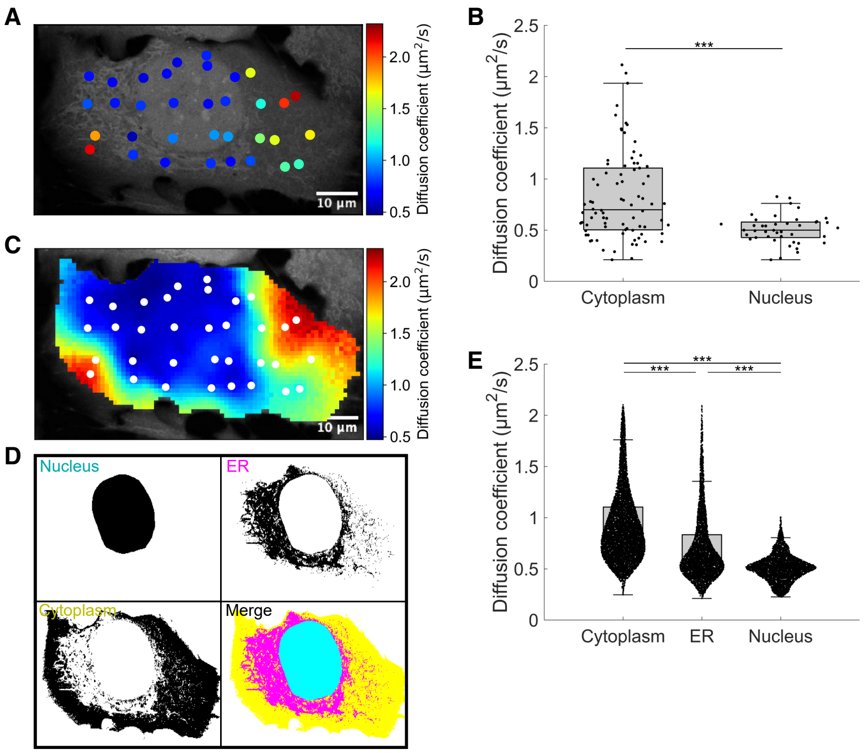

FIGURE 2 Subcellular analysis of diffusion heterogeneity. (*A*) Diffusion coefficients measured by FRAP. (*B*) Boxplots of the diffusion coefficients measured in the cytoplasm ($n = 82$) and nucleus ($n = 42$) from $N = 4$ independent experiments. (*C*) Diffusion coefficients determined by Pro-FRAP using the same dataset as in (*A*). White dots represent measurement points. (*D*) Classification of subcellular regions into the nucleus (*cyan*), ER (*magenta*), and the remaining cytoplasmic regions (*yellow*). (*E*) Boxplots of the diffusion coefficients for the three classified regions, obtained from the same dataset as in (*B*), based on $N = 4$ independent experiments.

and realistic cellular geometries (Figs. 3 and 4). We examined two distinct sampling strategies. In the first case, photobleached positions were determined using a Gaussian function with varying standard deviations σ, centered within the cell, allowing for a gradual decrease in measurement density from the center to the periphery (Fig. 3, *A–C*). To characterize these patterns in relation to cellular morphology, an equivalent cell radius R_{cell} was introduced by approximating the cell as a circle. In the second case, measurement points were arranged in a lattice pattern, ensuring a more uniform spatial coverage across the entire cell (Fig. 3 *D*). The diffusion coefficients were initially modeled as a continuous radial gradient, where values gradually increased from the center to the periphery (Fig. 3, *A–D*). SGS-estimated values were compared with known ground truths, with MAE and RMSE serving as evaluation metrics. Repeated simulations demonstrated that photobleaching patterns significantly influenced reconstruction accuracy (Fig. 3 *E*). Specifically, prediction errors progressively decreased as σ increased, reaching a plateau when σ/R_{cell} exceeded 0.5, a value comparable with half the cell radius. When σ was smaller, estimation accuracy declined, likely due to insufficient measurement coverage near the periphery. Similarly, a lattice pattern with a spacing comparable with the σ, which corresponds to half the cell radius, improved accuracy (Fig. 3 *E*). Similar trends were observed across different cell morphologies, although elongated cells exhibited greater error variability than rounded ones (Fig. 4, *A–C*).

To further investigate the effect of measurement density, simulations were conducted under two conditions: ordinary kriging alone and a combination of ordinary kriging with SGS. These analyses were performed using a Gaussian-distributed measurement pattern at $\sigma/R_{cell} = 0.56$, with the same cellular morphology and ground truth diffusion coefficient distribution as in Fig. 3 *A*. In ordinary kriging, errors monotonically decreased with increasing measurement points due to its linear interpolation nature, resulting in progressively improved prediction accuracy (Fig. S9). In contrast, although SGS also exhibited error reduction with increasing measurement density, the errors did not converge to zero, while the variability of the estimated values systematically decreased (Fig. 3 *F*). Because SGS sequentially incorporates estimated values as known data, its performance becomes increasingly stable when the initial predictions are accurate. When an optimal FRAP pattern effectively reflects spatial characteristics in the early stages of estimation, subsequent predictions remain reliable, reducing the overall dependence on the number of measurement points. Conversely, suboptimal sampling patterns can introduce spatial inaccuracies that propagate through the sequential estimation process, potentially affecting prediction stability.

In addition, when diffusion coefficients were modeled as a stepwise concentric pattern, where values exhibited discrete transitions rather than a continuous gradient, accuracy stabilized when σ/R_{cell} reached ~0.5 (Fig. 4, *D–F*), whereas smaller σ/R_{cell} values resulted in larger errors (Fig. S10). These similar trends suggest that the observation

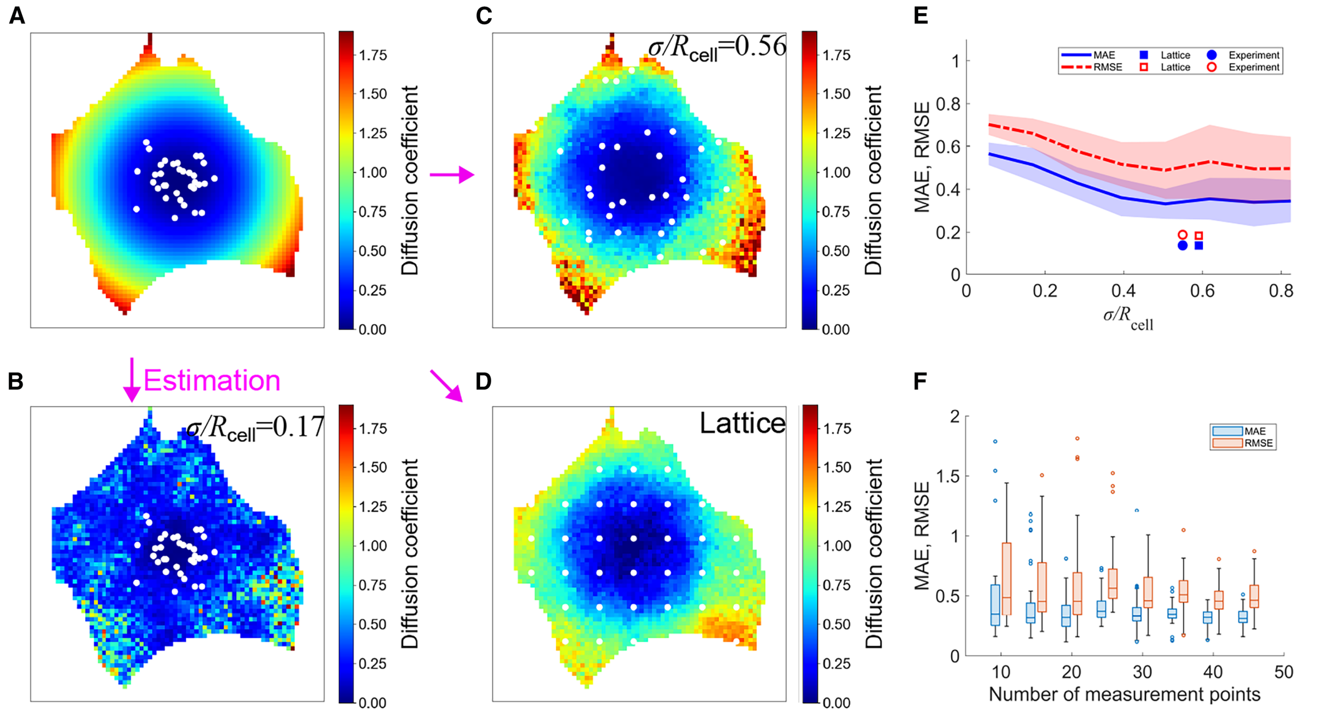

FIGURE 3 Numerical simulation to evaluate the process of Pro-FRAP. (*A*) Diffusion coefficients were assigned in a concentric pattern, and measurement points (*white dots*) were distributed according to a Gaussian function. (*B* and *C*) Diffusion coefficients determined by Pro-FRAP at $\sigma/R_{cell} = 0.17$ (*B*) and $\sigma/R_{cell} = 0.56$ (*C*). (*D*) Diffusion coefficients determined by Pro-FRAP using the same given diffusion distribution as in (*A*), but with measurement points (*white dots*) arranged in a lattice pattern. (*E*) Relationship between errors and σ/R_{cell}, where the mean and standard deviation of MAE (*blue*) and RMSE (*red*) are shown. The corresponding σ/R_{cell} values for the lattice-based analysis (*squares*) and those used in actual experimental analysis (*circles*) are also plotted. (*F*) Effect of increasing the number of measurement points in SGS analysis on errors (MAE in *blue* and RMSE in *red*) when σ/R_{cell} is fixed at 0.56.

remains consistent across different conditions. Accuracy thus consistently stabilized when σ/R_{cell} exceeded ~0.5, corresponding to one-fourth of the cell size. Because a Gaussian distribution extends beyond its standard deviation, measurement points need to cover peripheral regions to maintain accuracy. Based on these findings, we adopted ~30 measurement points per cell in actual experiments, ensuring comprehensive spatial coverage by distributing photobleaching points as evenly as possible.

DISCUSSION

In this study, we developed Pro-FRAP, a novel framework integrating FRAP with SGS, a powerful spatial statistical method, for estimating molecular diffusion across unmeasured regions. Traditional FRAP techniques, with their localized measurement approach, are inherently limited by excessive photobleaching, which compromises measurement accuracy. Consequently, capturing the full complexity of molecular dynamics within the cellular context becomes challenging. This limitation is particularly significant in analyzing spatial variation given that intracellular macromolecular crowding inherently creates heterogeneity (16). By overcoming these constraints, our approach opens the possibility of statistically linking whole-cell dynamics with molecular regulation, beyond what localized measurements alone can reveal. Importantly, our proposed framework is not intended to replace conventional FRAP or any other existing experimental techniques. Instead, it introduces a general conceptual framework for applying spatial statistical methods to intracellular measurements and complements existing techniques that provide spatially limited measurements by enabling researchers to interpolate unmeasured regions and quantify uncertainties.

Spatial statistics have been extensively utilized in various fields including geology, environmental science, and epidemiology to analyze spatially correlated data. These techniques enable the estimation of values in unmeasured regions based on the spatial correlation of measured data points. In our study, we applied the spatial statistical method SGS to analyze intracellular diffusion. As a probabilistic simulation method, SGS enables the quantification of data uncertainty and generates multiple realizations, allowing for a more comprehensive assessment of spatial variability. Our results demonstrated its effectiveness in enabling spatially continuous reconstruction of intracellular diffusion landscapes.

In spatial statistics, unmeasured regions are treated as random variables, enabling statistically robust estimation based on measured data. This approach not only provides a solid statistical foundation but also incorporates spatial autocorrelation, ensuring that predictions align with locally

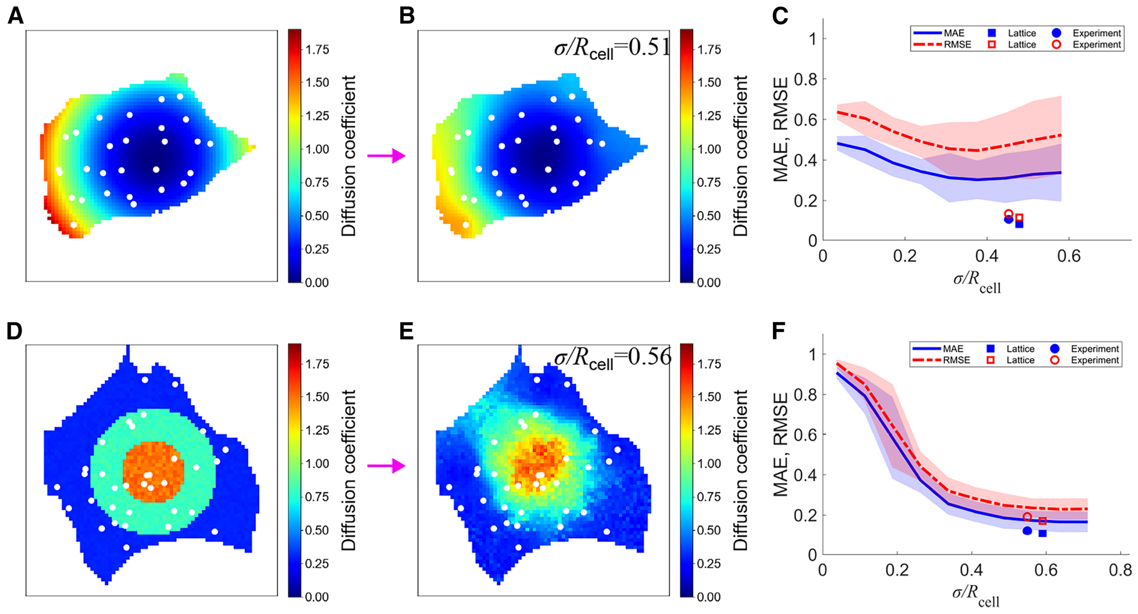

FIGURE 4 Additional numerical simulation evaluating the performance of Pro-FRAP. (*A*) Given diffusion coefficient pattern with a continuous concentric gradient and the distribution of measurement points (*white*). (*B*) Diffusion coefficients determined by Pro-FRAP with a Gaussian-distributed measurement pattern at $\sigma/R_{cell} = 0.51$. (*C*) Relationship between errors and σ/R_{cell}, where the mean and standard deviation of MAE (*blue*) and RMSE (*red*) are shown. The corresponding σ/R_{cell} values for lattice-based analysis (*squares*) and those used in actual experimental analysis (*circles*) are also plotted. (*D*) Given diffusion coefficient pattern with stepwise concentric transitions and the distribution of measurement points (*white*). (*E*) Diffusion coefficients determined by Pro-FRAP with a Gaussian-distributed measurement pattern at $\sigma/R_{cell} = 0.56$. (*F*) Relationship between errors and σ/R_{cell}, where the mean and standard deviation of MAE (*blue*) and RMSE (*red*) are shown. The corresponding σ/R_{cell} values for lattice-based analysis (*squares*) and those used in actual experimental analysis (*circles*) are also plotted.

observed data. In contrast, some alternative methods might attempt to determine values in unmeasured regions through deterministic means such as the use of spline functions for interpolation. These methods lack the rigorous statistical justification that spatial statistical techniques offer. By combining the optimal linear unbiased predictions of kriging with the ability of SGS to generate multiple realizations of spatial fields, spatial statistical methods capture the full range of spatial variability while providing statistically grounded predictions.

In practical applications, the choice between ordinary kriging and SGS depends on the balance between computational cost and the need for uncertainty quantification. Ordinary kriging analytically provides a single optimal estimate based on the fitted spatial model, making it suitable when rapid approximate mapping is sufficient and detailed uncertainty estimates are not required. In contrast, SGS generates multiple realizations to quantify local uncertainties and better capture spatial variability, but it is computationally more demanding. In the current case, approximately 100 SGS iterations were sufficient for convergence under our experimental conditions (Fig. S6). Thus, ordinary kriging can be a practical alternative when computational resources are limited, whereas SGS is preferable when evaluating local uncertainty or capturing spatial variability is critical.

Other intracellular diffusion measurements such as FCS often face the same limitation due to their localized measurement nature. While FCS can be strengthened by combining high-speed acquisition to cover larger areas (17), this approach is not feasible for FRAP because extensive photobleaching results in loss of fluorescence signal across the cell. Recent studies have developed the use of small fluorescent particles or genetically encoded multimeric (GEM) nanoparticles to analyze the diffusion coefficient across whole cells (18). However, this method requires customized GEM nanoparticles and is therefore not suitable for analyzing the diffusion of specific target molecules. Alternative techniques such as raster image correlation spectroscopy (19), spatiotemporal image correlation spectroscopy (20), and superresolution optical fluctuation imaging (21) offer spatially resolved diffusion measurements, yet they typically require careful adjustment of acquisition parameters depending on the molecular dynamics and imaging system. Superresolution optical fluctuation imaging enhances spatial resolution using fluorescence fluctuations but is not designed to directly quantify diffusivity, and typically requires blinking fluorophores with specific photophysical properties. Our approach circumvents or overcomes these limitations by enabling spatial mapping of intracellular diffusivity for arbitrary fluorescent species without the need for specialized probes or instrumentation.

An important consideration in spatial modeling is the attainable resolution of the reconstructed diffusivity map.

In our study, the raw resolution was defined by the FRAP support size (∼3 μm), which determines the spatial extent over which diffusion is averaged. However, the effective resolution depends on the spatial correlation of diffusivity values across the cell. An estimate of the effective resolution can be obtained from the semivariogram of the reconstructed diffusivity field (Fig. 1 *F*), as the onset of its rise above the noise floor typically occurs around 10 μm, indicating that spatial inhomogeneities at or above this scale are detectable. For reference, geostatistical practice often considers the smallest resolvable scale to be roughly 1/5–1/6 of the variogram range (typically 30–40 μm in our case), yielding a resolution estimate of 5–7 μm. This serves as a practical lower bound for resolvable spatial features.

Although FRAP is traditionally used to measure diffusion, it can also provide insights into other dynamic processes such as chemical binding and dissociation, namely turnover, as well as macroscopic deformations and advection (22,23). Although we focused herein only on the diffusion of a specific compound to evaluate the usefulness of our approach, the combination of FRAP with spatial statistical techniques may be extended to analyze other physicochemical properties, which will be the subject of future research. In this regard, while the recently developed method using GEM nanoparticles is limited to diffusion measurement, our approach potentially covers more diverse applications, providing a more comprehensive understanding of the complex molecular behavior in cells.

The complexity of the intracellular environment necessitates advanced statistical methods for accurate analysis. Our separate analyses revealed that, while kriging alone achieves an average prediction accuracy of ∼75%, SGS displayed superior accuracy, achieving ∼85% (Fig. S5). Kriging optimally utilizes spatial correlations for predictions with well-defined statistical confidence, but its reliance on nearby observations can limit its ability to capture global variability. Indeed, increasing the number of measurement points consistently improved accuracy (Fig. S9), but this approach is impractical due to fluorescence bleaching constraints. In contrast, SGS effectively captures spatial variability with lower error fluctuation, although it requires a substantial computational load. Despite this, the advantages of SGS allowed us to achieve high-resolution, statistically reliable mapping of molecular diffusion within cells.

Recent advances in omics technologies and mass spectrometry, including single-cell RNA sequencing, mass spectrometry imaging, and spatial transcriptomics, have revolutionized the spatial mapping of intracellular components at a single-cell level, providing unprecedented insights into cellular spatial organization (24–26). Combining these cutting-edge technologies with the Pro-FRAP approach could offer new perspectives on how intracellular components and their physicochemical dynamics are functionally interconnected. Thus, the proposed method has the potential to deepen our understanding of cellular functions and their spatial regulation.

In conclusion, Pro-FRAP, which combines FRAP with spatial statistical analysis, provides a robust framework for visualizing molecular diffusion at the whole-cell level. This approach addresses the inherent limitations of traditional FRAP techniques, particularly those related to localized measurements and photobleaching. By integrating the advanced spatial statistics method SGS, we achieved statistically grounded mapping of molecular diffusion. This versatile framework is not inherently limited to diffusion mapping alone but can be extended to analyze other dynamic processes such as turnover, macroscopic deformations, and advection, ultimately providing deeper insights into the complex physicochemical behavior of intracellular molecules.

DATA AND CODE AVAILABILITY

The source codes of SGS are publicly available on GitHub (https://github.com/Deguchi-Lab-Osaka/cell-spacial-statistics).

ACKNOWLEDGMENTS

This study was partly supported by JSPS KAKENHI grants (21H03796, 22J00060, 23K26040, and 23H04929).

AUTHOR CONTRIBUTIONS

Y.O. performed the experiments with feedback from T.S. Y.O. analyzed the data with feedback from T.S. and D.M. O.N. conducted the SGS analysis. Y.O. and O.N. prepared the figures with feedback from T.S. and S.D. D.M. provided support for analysis and resources. S.D. led the design of the research and supervised its execution. T.S. and S.D. wrote the draft. All authors reviewed and approved the final manuscript.

DECLARATION OF INTERESTS

The authors declare no competing interests.

SUPPORTING MATERIAL

Supporting material can be found online at https://doi.org/10.1016/j.bpj.2025.10.005.

REFERENCES

1. Blonk, J. C. G., A. Don, …, J. J. Birmingham. 1993. Fluorescence photobleaching recovery in the confocal scanning light microscope. *J. Microsc.* 169:363–374.
2. Kenworthy, A. K. 2023. What's past is prologue: FRAP keeps delivering 50 years later. *Biophys. J.* 122:3577–3586.
3. Saito, T., D. Matsunaga, …, S. Deguchi. 2021. Determining the domain-level reaction-diffusion properties of an actin-binding protein transgelin-2 within cells. *Exp. Cell Res.* 404:112619.
4. Geiger, A. C., C. J. Smith, …, G. J. Simpson. 2020. Anomalous Diffusion Characterization by Fourier Transform-FRAP with Patterned Illumination. *Biophys. J.* 119:737–748.

5. Cowan, A. E., and L. M. Loew. 2023. Beyond analytic solution: Analysis of FRAP experiments by spatial simulation of the forward problem. *Biophys. J.* 122:3722–3737.
6. Saito, T., K. Kikuchi, and T. Ishikawa. 2024. Glucose stockpile in the intestinal apical brush border in C. elegans. *Biochem. Biophys. Res. Commun.* 706:149762.
7. Walser, M., A. Hajnal, and J. M. Escobar-Restrepo. 2015. FRAP analysis of LET-23::GFP in the vulval epithelial cells of living *Caenorhabditis elegans* larvae. *Bio-Protocol.* 5:e1489.
8. Dross, N., C. Spriet, …, J. Langowski. 2009. Mapping eGFP oligomer mobility in living cell nuclei. *PLoS One.* 4:e5041.
9. Kühn, T., T. O. Ihalainen, …, J. Timonen. 2011. Protein diffusion in mammalian cell cytoplasm. *PLoS One.* 6:e22962.
10. Talesh Hosseini, S., O. Asghari, and S. R. Ghavami Riabi. 2018. Spatial modelling of zonality elements based on compositional nature of geochemical data using geostatistical approach: a case study of Baghqloom area, Iran. *J. Min. Environ.* 9:153–167.
11. Bai, T., and P. Tahmasebi. 2022. Sequential Gaussian simulation for geosystems modeling: A machine learning approach. *Geosci. Front.* 13:101258.
12. Goovaerts, P. 1999. Geostatistics in soil science: state-of-the-art and perspectives. *Geoderma.* 89:1–45.
13. Jonasson, J. K., N. Lorén, …, M. Rudemo. 2008. A pixel-based likelihood framework for analysis of fluorescence recovery after photobleaching data. *J. Microsc.* 232:260–269.
14. Jonasson, J. K., J. Hagman, …, M. Rudemo. 2010. Pixel-based analysis of FRAP data with a general initial bleaching profile. *J. Microsc.* 239:142–153.
15. Webster, R., and M. A. Oliver. 2008. Geostatistics for Environmental Scientists, Second Edition. John Wiley & Sons.
16. Buenaventura, A., T. Saito, …, S. Deguchi. 2024. Intracellular Macromolecular Crowding within Individual Stress Fibers Analyzed by Fluorescence Correlation Spectroscopy. *Cell. Mol. Bioeng.* 17:165–176.
17. Scipioni, L., M. Di Bona, …, L. Lanzanò. 2018. Local raster image correlation spectroscopy generates high-resolution intracellular diffusion maps. *Commun. Biol.* 1:10.
18. Delarue, M., G. P. Brittingham, …, L. J. Holt. 2018. mTORC1 Controls Phase Separation and the Biophysical Properties of the Cytoplasm by Tuning Crowding. *Cell.* 174:338–349.e20.
19. Rossow, M. J., J. M. Sasaki, …, E. Gratton. 2010. Raster image correlation spectroscopy in live cells. *Nat. Protoc.* 5:1761–1774.
20. Hebert, B., S. Costantino, and P. W. Wiseman. 2005. Spatiotemporal image correlation spectroscopy (STICS) theory, verification, and application to protein velocity mapping in living CHO cells. *Biophys. J.* 88:3601–3614.
21. Basak, S., A. Chizhik, …, J. Enderlein. 2025. Super-resolution optical fluctuation imaging. *Nat. Photonics.* 19:229–237.
22. Saito, T., D. Matsunaga, and S. Deguchi. 2022. Analysis of chemomechanical behavior of stress fibers by continuum mechanics-based FRAP. *Biophys. J.* 121:2921–2930.
23. Saito, T., D. Matsunaga, and S. Deguchi. 2022. Long-term molecular turnover of actin stress fibers revealed by advection-reaction analysis in fluorescence recovery after photobleaching. *PLoS One.* 17: e0276909.
24. Rodriques, S. G., R. R. Stickels, …, E. Z. Macosko. 2019. Slide-seq: A scalable technology for measuring genome-wide expression at high spatial resolution. *Science.* 363:1463–1467.
25. Giesen, C., H. A. O. Wang, …, B. Bodenmiller. 2014. Highly multiplexed imaging of tumor tissues with subcellular resolution by mass cytometry. *Nat. Methods.* 11:417–422.
26. Longo, S. K., M. G. Guo, …, P. A. Khavari. 2021. Integrating single-cell and spatial transcriptomics to elucidate intercellular tissue dynamics. *Nat. Rev. Genet.* 22:627–644.

Biophysical Journal, Volume *124*

Supplemental information

Mapping intracellular dynamics across the whole cell with spatial statistics

Yohei Okabe, Takumi Saito, Outa Nakashima, Daiki Matsunaga, and Shinji Deguchi

Supporting materials for Okabe et al., "Mapping intracellular dynamics across the whole cell with spatial statistics"

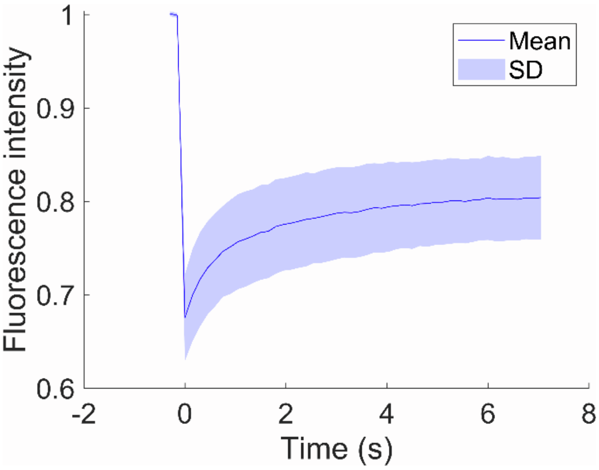

Figure S1. Representative FRAP recovery curve. Fluorescence intensity is normalized to the pre-bleach value ($t = 0$ s). The solid line represents the mean, and the shaded area indicates the standard deviation from $n = 18$ data points within a single cell.

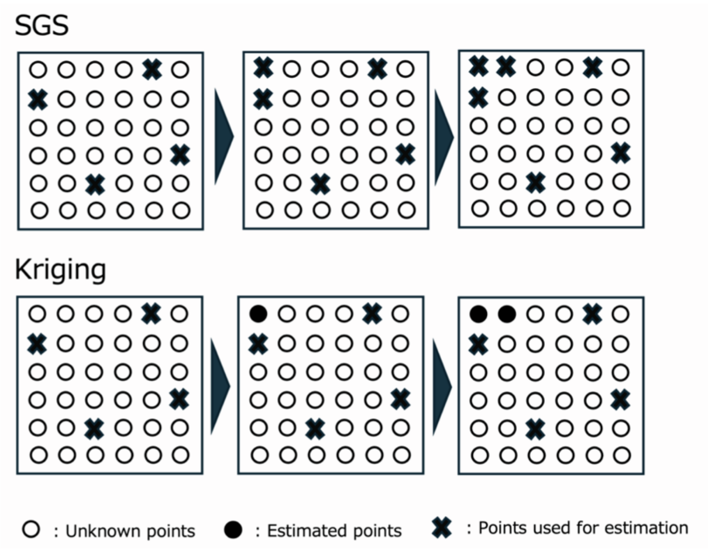

Figure S2 Schematic diagram of SGS and kriging. In SGS, estimation is performed by sequentially incorporating newly estimated data into the known dataset. In contrast, kriging uses only the originally available known data for estimation.

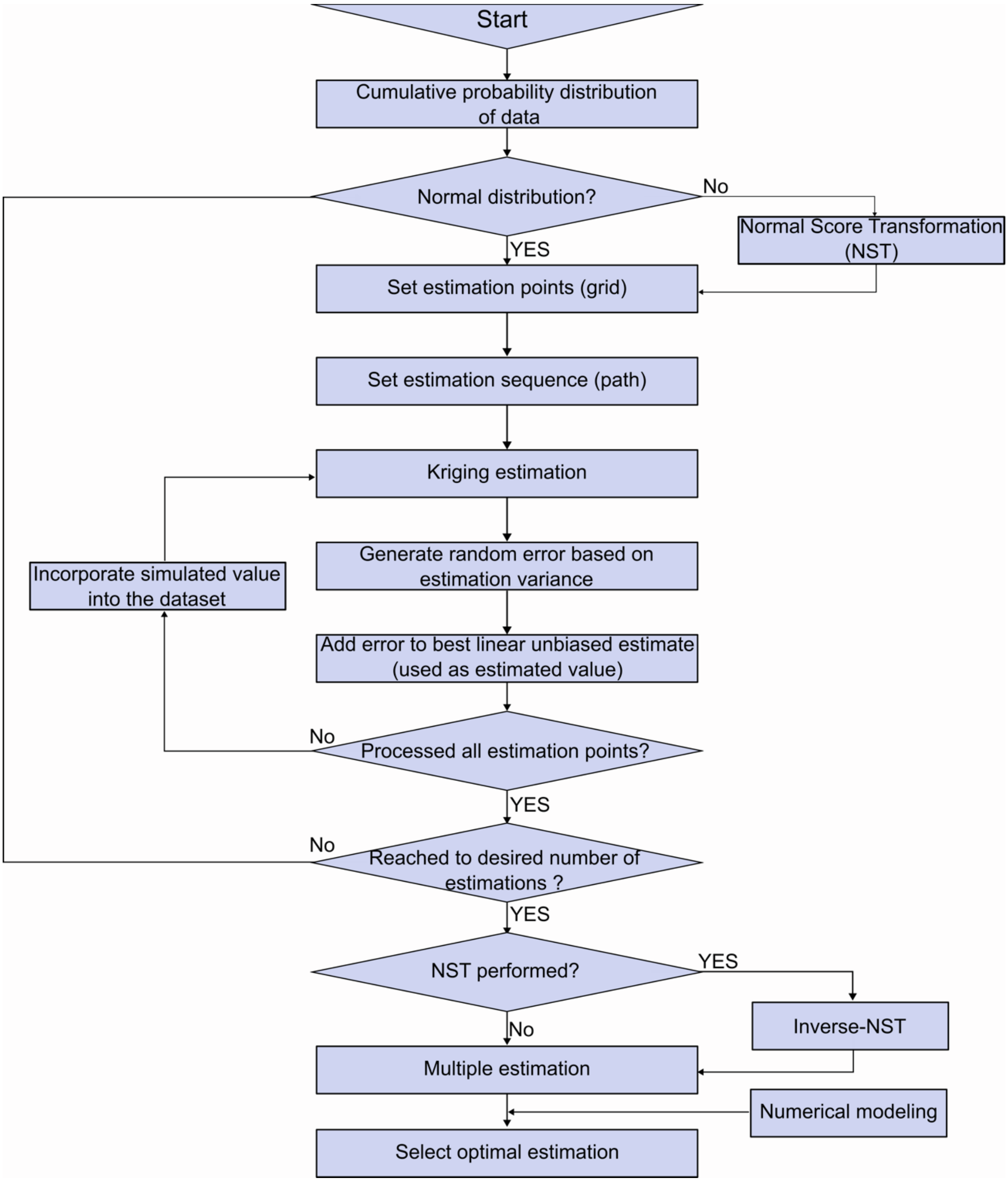

Figure S3 SGS realization used for diffusivity estimation. The diagram illustrates the step-by-step workflow from data transformation and grid setup to sequential kriging with random error incorporation, repeated for multiple realizations to quantify spatial variability.

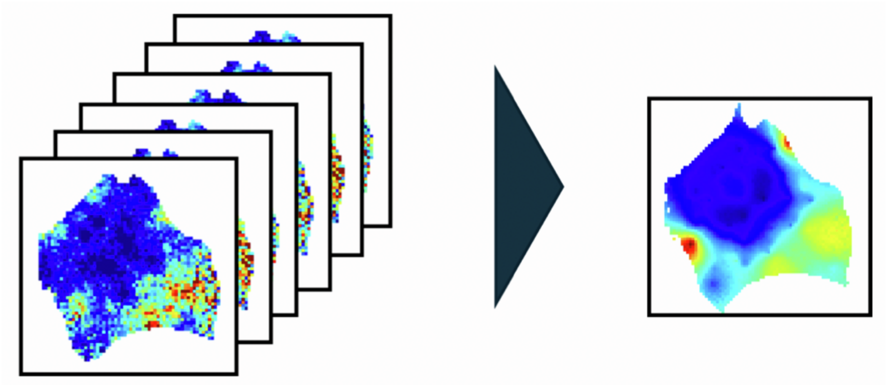

Figure S4 Schematic diagram of SGS simulation averaging. The final estimated value is obtained by averaging the results of multiple simulations.

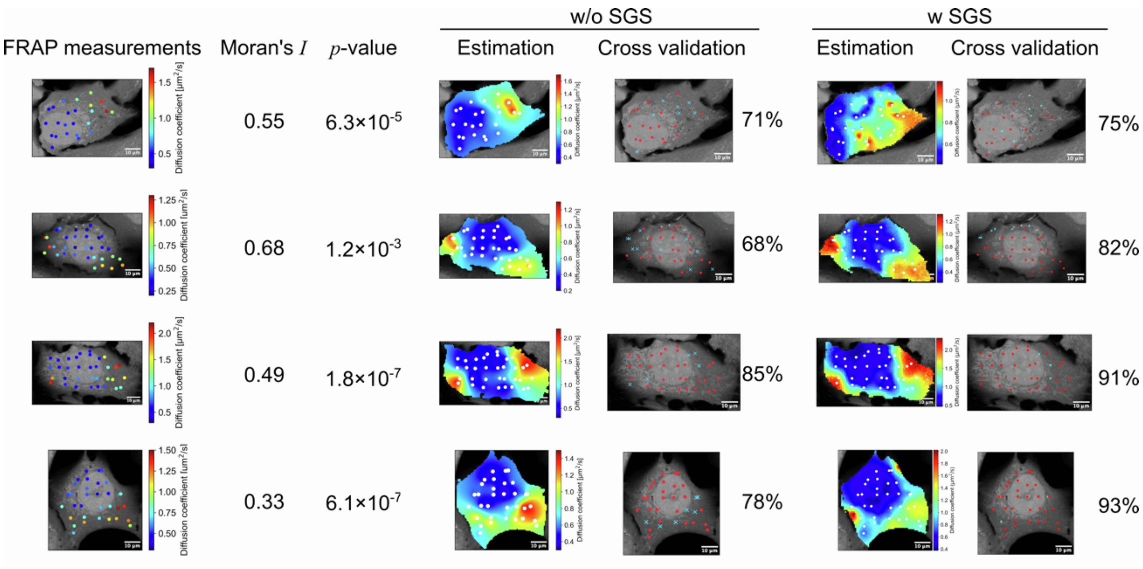

Figure S5 Validation of Pro-FRAP using four representative datasets. The left column shows the diffusion coefficients measured by FRAP, along with the corresponding Moran's I and p-values. The middle column (w/o SGS) and the right column (w SGS) display the diffusion coefficient distributions determined using kriging alone (middle) and Pro-FRAP with SGS (right), respectively. Cross-validation results are also shown, where correctly predicted points are marked in red, and incorrectly predicted points defined as those deviating by more than one standard deviation from the normal distribution are indicated by blue crosses.

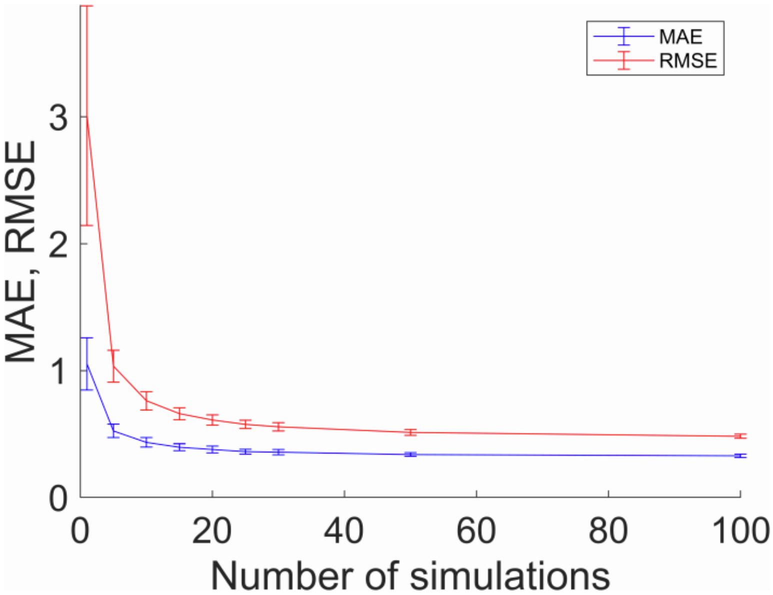

Figure S6 Effect of the number of SGS iterations on error convergence. Both MAE and RMSE sufficiently converge by n = 100.

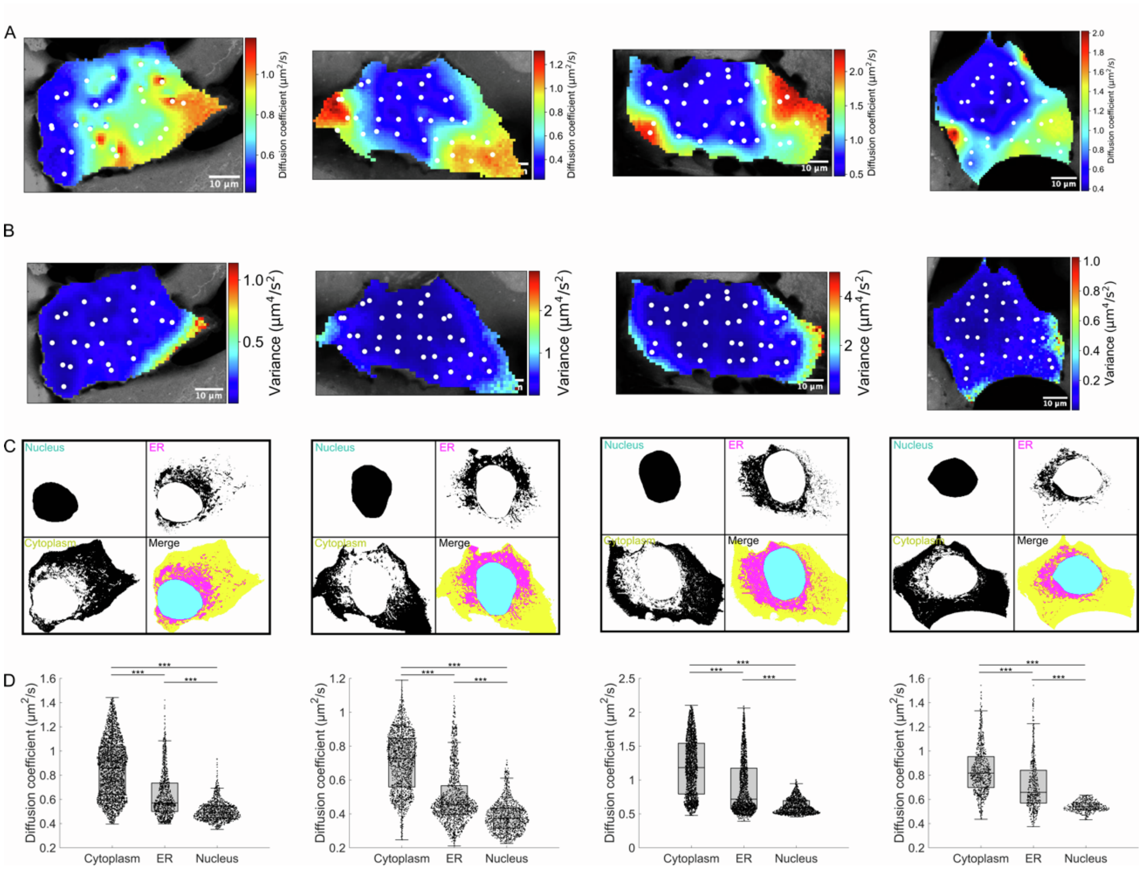

Figure S7 Subcellular analysis of diffusion coefficients for four representative datasets. (A, B) Mean diffusion coefficients (A) and variance (B). (C) Segmentation of subcellular regions into the nucleus, ER, and remaining cytoplasm. (D) Diffusion coefficients for each classified region.

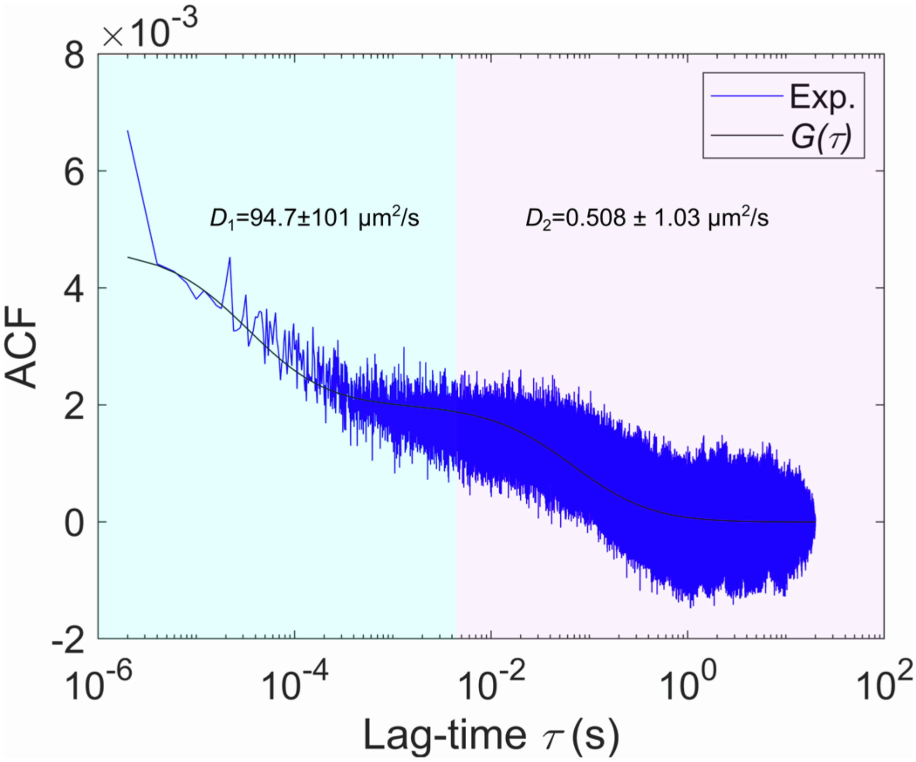

Figure S8 Representative FCS autocorrelation curve and dual-component fitting. ACF derived from a fluorescence intensity time series (blue) with an overlaid fit using a dual-component diffusion model (black line). The fast and slow components correspond to distinct diffusion regimes, as indicated by the shaded cyan and magenta regions, respectively. The slow component is likely due to complex formation following covalent binding of the dye to thiol-containing macromolecules after esterase activation.

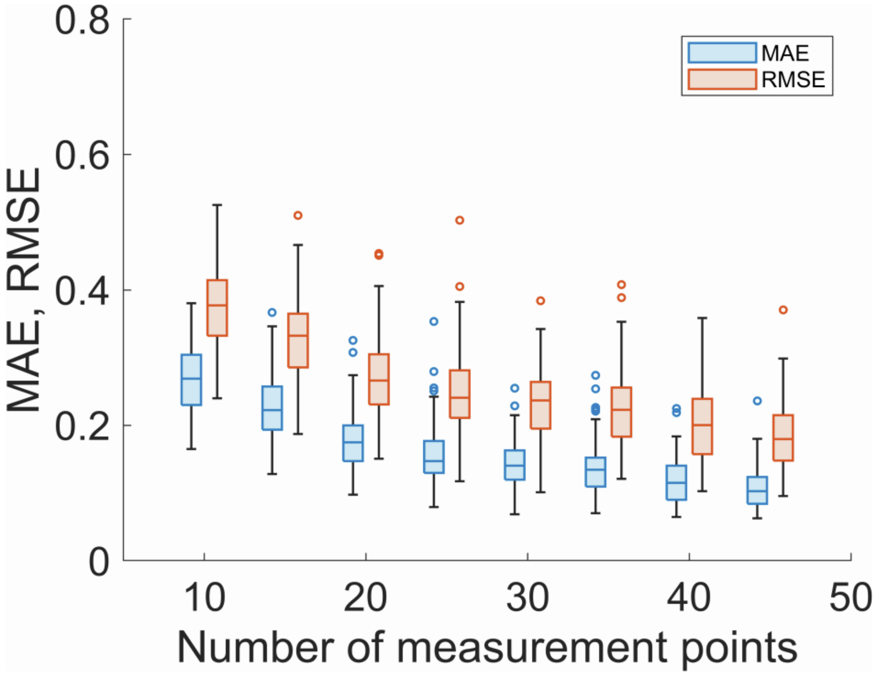

Figure S9 Evaluation of ordinary kriging. Effect of increasing the number of measurement points in ordinary kriging alone on errors (MAE in blue and RMSE in red) when σ/R_{cell} is fixed at 0.56.

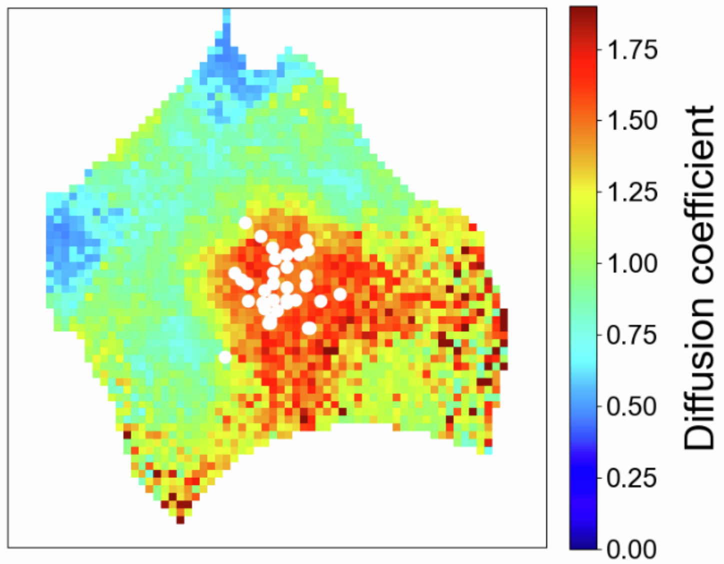

Figure S10 Additional analysis of Fig. 4D. Diffusion coefficients determined by Pro-FRAP with a Gaussian-distributed pattern of σ/R_{cell} = 0.17, corresponding to Fig. 4D.

Supporting note 1

Ordinary kriging assumes a steady state in the spatial domain, and the mean of a physical variable m is constant but unknown. The unbiasedness condition for predictions is expressed as

$$\sum_{k=1}^{n} \omega_k = 1 \tag{S1}$$

Here, ω_k represents the weights assigned to the observed data positions u_k. The prediction error variance of a variable Z relative to the true value is expressed as

$$\begin{aligned}
\sigma_\varepsilon^{2*} &= V[\, Z^*(\, u_o) - Z(\, u_o)] \\
&= V[\textstyle\sum_{k=1}^{n} \omega_k \, Z(u_k) - Z(u_o)] \\
&= V\left[\textstyle\sum_{k=1}^{n} \omega_k \left(Z(u_k) - Z(u_o)\right)\right] \\
&= E[\{\textstyle\sum_{k=1}^{n} \omega_k (Z(u_k) - Z(u_o))\}^2] \\
&= \textstyle\sum_{j=1}^{n} \sum_{k=1}^{n} \omega_j \omega_k E\left[\left(Z(u_j) - Z(u_o)\right)\left(Z(u_k) - Z(u_o)\right)\right]
\end{aligned} \tag{S2}$$

where V and E represent the variance and expected value, respectively; u_o and u_k denote the positions of an unobserved point and of an arbitrary point, respectively; and an asterisk indicates estimated variables. The semi-variogram is defined as half of the squared difference between values at any given distance h:

$$\gamma(h_{j,k}) = \frac{1}{2} V\left[Z(u_j) - Z(u_k)\right] = \frac{1}{2} E\left[\left(Z(u_j) - Z(u_k)\right)^2\right]$$

$$= \frac{1}{2} E\left[\left(Z(u_j) - Z(u_o)\right)^2\right] + \frac{1}{2} E[(Z(u_k) - Z(u_o))^2] - E\left[\left(Z(u_j) - Z(u_o)\right)(Z(u_k) - Z(u_o))\right]$$

$$= \gamma(h_{o,j}) + \gamma(h_{o,k}) - E\left[\left(Z(u_j) - Z(u_o)\right)(Z(u_k) - Z(u_o))\right] \quad .$$

(S3)

Substituting Eq. (S3) into Eq. (S2) and eliminating E,

$$\sigma_\varepsilon^{2*} = \sum_{j=1}^{n}\sum_{k=1}^{n}\omega_j\omega_k\left(\gamma(h_{o,j}) + \gamma(h_{o,k}) - \gamma(h_{j,k})\right)$$

$$= \sum_{j=1}^{n}\omega_j\gamma(h_{o,j}) + \sum_{k=1}^{n}\omega_k\gamma(h_{o,k}) - \sum_{j=1}^{n}\sum_{k=1}^{n}\omega_j\omega_k\gamma(h_{j,k})$$

$$= 2\sum_{k=1}^{n}\omega_k\,\gamma(h_{o,k}) - \sum_{j=1}^{n}\sum_{k=1}^{n}\omega_j\omega_k\gamma(h_{j,k}). \quad \text{(S4)}$$

We consider a Lagrange multiplier problem to minimize the semi-variogram under the constraint given by Eq. (S1),

$$Q = \frac{1}{2}\,\sigma_\varepsilon^{2*} + \mu\left(1 - \sum_{k=1}^{n}\omega_k\right). \quad \text{(S5)}$$

The variables ω_k and μ are determined by minimizing the objective function Q, and thus

$$\frac{\partial Q}{\partial \omega_k} = \gamma(h_{j,o}) - \sum_{k=1}^{n}\omega_k\gamma(h_{j,k}) - \mu = 0 \quad \text{(S6)}$$

and

$$\frac{\partial Q}{\partial \mu} = 1 - \sum_{k=1}^{n}\omega_k = 0 \quad \text{(S7)}$$

The combination of Eqs. (S6) and Eq. (S7) forms the kriging equations (i.e., Eq. (3) and (4) in the main text), which are a system of $n+1$ linear equations with unknowns ω_k

$(k = 1, 2, \ldots, n)$ and μ.
